# Compulsory licensing of trade secrets: ensuring access to COVID-19 vaccines via involuntary technology transfer

**DOI:** 10.1093/jiplp/jpab129

**Published:** 2021-12-01

**Authors:** Olga Gurgula, John Hull

**The authors**

- Dr Olga Gurgula (PhD, LLM) is a Senior lecturer in Intellectual Property Law at Brunel Law School, Brunel University London. Mr John Hull (LLM) is Visiting Professor at King’s College London, Teaching Fellow at Queen Mary, University of London and a practising lawyer in the UK.

**This article**

- This paper considers how vaccine technology to meet the challenges of the COVID-19 pandemic can be made available to increase the production of vaccines. Its primary focus is on trade secrets, which are one of the main intellectual property (IP) rights protecting the complex manufacturing processes of vaccine production.
- The compulsory licensing of trade secrets presents some unique obstacles, and consideration is given to some practical solutions that might balance the interests of technology owners and the public interest in increased access to vaccines. In particular, this paper suggests that to make the currently discussed proposals on accelerating the production of COVID-19 vaccines, including compulsory licensing of patents and the TRIPS IP waiver, work, an additional mechanism of compulsory licensing of trade secrets is required.
- It is believed that a proposal for a new mechanism of compulsory licensing of trade secrets coupled with a discussion on the content of such licences, the challenges that would need to be addressed and the potential wording of such a licence would provide useful guidance to governments on how to make their compulsory technology transfer mechanisms more effective.

## 1. Introduction

On 30 March 2021, 25 heads of government and international agencies came together in an extraordinary joint call for a new international treaty for pandemic preparedness and response

There will be other pandemics and other major health emergencies. No single government or multilateral agency can address this threat alone. The question is not if, but when.^1^

As the world has been battling the coronavirus (COVID-19) pandemic, this call reflects the gloomy reality of the challenges that this pandemic has brought to us and the inadequacy of the current system to deal with it. While the first enormous challenge of swiftly developing a vaccine against this coronavirus has been successfully overcome by several pharmaceutical companies^2^ and a number of vaccines are in the pipeline at various stages of development,^3^ the second, no less significant hurdle, is to manufacture the required number of vaccines and distribute them across the globe equitably and affordably. However, this latter hurdle has proven to be a serious challenge. It is estimated that around 11 billion doses are required to vaccinate 70 per cent of the world’s population.^4^ According to the World Health Organisation (WHO), as of 5 May 2021, more than 1.1 billion doses of vaccine have been administered globally, but more than 80 per cent of those have been administered in high- and upper-middle-income countries, with only 0.3 per cent in low-income countries.^5^ It is argued that it may take several years for people in the lowest-income groups to be vaccinated.^6^ This problem, therefore, has raised a key question, i.e. how can we accelerate the production of COVID-19 vaccines and ensure their equitable worldwide distribution at an affordable price?

## 2. Access to vaccine technology

While the COVID-19 pandemic has escalated the problem of access to vaccines, long before this pandemic, it was recognized that access to vaccines in low- and middle-income countries (‘LMICs’) lagged far behind access in higher-income countries.^7^ To remedy this, access to manufacturing information protected by trade secrets and know-how held by pharmaceutical companies is necessary. One option to provide such access is through patent pools and technology transfer ‘hubs’ of the sort devised by the WHO in 2007 as a means of increasing the number of influenza vaccine producers in LMICs, which has had some success.^8^ Nevertheless, a patent pool or a technology transfer hub is ill suited for accessing manufacturing trade secrets and associated know-how.^9^ However innovative, such an approach could only be successful if the technology owners bought into the idea and were willing to disclose their trade secret process information to the patent pool. The question is: why should they? The risk of loss of secrecy in the process is simply too great to induce any of them to participate voluntarily. It is clear that trade secrets are seen by pharmaceutical companies as particularly valuable intellectual property (IP) rights.^10^ This is evident from the developments around the COVID-19 Technology Access Pool (‘C-TAP’), which was set up by the WHO in May 2020.^11^ It calls the global community to action, and most importantly pharmaceutical companies, to voluntarily share knowledge, IP and data necessary to defeat COVID-19.^12^ However, this initiative has attracted zero contributions since it was established, as pharmaceutical companies refuse to share their vaccine technologies with this and similar initiatives.^13^ It is now increasingly recognized that there is no mechanism in IP laws to oblige trade secret owners to share their technology.^14^

Another option for removing the barriers to the timely provisioning of affordable COVID-19 medical products was suggested in October 2020 by South Africa and India.^15^ In the revised proposal submitted in May 2021, they requested that the World Trade Organisation waive certain provisions of the Trade-Related Aspects of Intellectual Property Rights (‘TRIPS’) Agreement^16^ for the prevention, treatment or containment of COVID-19, including patents and undisclosed information regarding vaccines and related health technologies; such a waiver is proposed to be in force for at least 3 years from the date of the decision.^17^ That request has gathered pace and been given additional force with the support of the Biden administration in the USA (albeit for COVID-19 vaccines only).^18^ It is important to appreciate what the IP waiver is. At the WTO level, if the waiver is agreed upon, WTO members would not be able to sue a WTO member for TRIPS non-compliance in the case where it waives IP rights at a national level. The effect of the IP waiver at a national level, in turn, is that by implementing the IP waiver, IP rights would not be enforceable against third parties. Specifically, the implementation of the IP waiver would presuppose suspending the enforceability of a number of IP rights and declaring that, during the period of the IP waiver, the manufacture of the IP-protected products (and other activities that fall within the exclusive rights of the IP owner) by third parties without the permission of the IP rights holder would not be considered an infringement.

A number of WTO members, including the European Union (EU), Norway, the UK, and Switzerland, however, oppose this waiver, claiming, among other things, that the current TRIPS flexibilities, such as compulsory licensing, are sufficient and could be used to deal with IP-related barriers concerning vaccines.^19^

While the IP waiver or compulsory licensing of patents may help in accelerating the production of vaccines, these mechanisms have one significant drawback. Vaccines are complex biologics, and their manufacture is challenging because of, inter alia, the special facilities and equipment needed, complex processes involved and the specialist knowledge and experience required.^20^ Such knowledge is typically protected by patents and, more importantly, by trade secrets. This has prompted a fierce debate about whether pharmaceutical companies should share their IP-protected technology with others. Some commentators suggest that ‘arguments to defend IP rights simply do not hold’,^21^ referring to the fact that much COVID-19 vaccine research was done with public funding and on that basis call for a waiver not just on patents but on trade secrets, manufacturing know-how, industrial design, blueprints and so on. Others claim that ‘trade secrets are not sacrosanct’ and on that basis they appeal for disclosure on public interest grounds.^22^ However, even if we start from the premise that trade secrets should be shared, another challenging question is how should ‘trade secret sharing’ or their compulsory licensing be carried out? More specifically, how might this be achieved in a way that balances the needs of the public and fairness to trade secret rights holders whose fragile rights are to be put into the hands of third-party licensees? In this respect, most of the proponents of the IP waiver, compulsory licensing or enforced technology transfer are notably silent.^23^

The aim of this paper, therefore, is to suggest certain solutions intended to accelerate the production of vaccines by focusing on the mechanism of compulsory licensing of vaccine technologies. While the current body of literature extensively discusses compulsory licensing of patents that protect vaccines,^24^ there is currently very limited discussion about trade secrets that protect vaccines and the need to share such information in order to produce a vaccine.^25^ This paper, therefore, aims to fill this gap by considering how trade secrets can be licensed and whether, in practice, the difficulties associated with this form of technology transfer are capable of being overcome.

The paper will briefly explain what a vaccine is and how it is typically protected by IP rights and data/marketing exclusivity. It will then explain why the current mechanism of compulsory licensing of patents is not sufficient to compulsorily licence a vaccine technology because vaccines are generally protected not only by patents but also by trade secrets for which there is no equivalent compulsory licensing mechanism. It will be suggested that to enforce an involuntary technology transfer for the production of a vaccine, governments must grant a complex/hybrid compulsory licence that would include not only patents but also their associated trade secrets. For this, it will be argued that compulsory licensing of trade secrets should be implemented in national and international IP laws to supplement the existing mechanism of compulsory licensing of patents. The paper will explain that the suggested mechanism of compulsory licensing of trade secrets is in line with international law related to trade secrets. It will also discuss a specific set of elements that would need to be considered when granting a compulsory licence of trade secrets so that this mechanism could work effectively and will provide suggestions on the specific wording of such a compulsory licence. The paper will conclude with some further considerations that governments may face when considering compulsory licensing of vaccine technologies, i.e. the need to implement exceptions from data and marketing exclusivity when granting a compulsory licence (both of patents and/or trade secrets).

It is important to note that the suggested mechanism of compulsory licensing of trade secrets would be useful both in case of implementation of the IP waiver by the World Trade organisation and if no such waiver were to be agreed upon. This is because in either scenario, access to trade secrets related to vaccine manufacture would be required, and if pharmaceutical companies did not voluntarily share such information potential manufacturers would require developing their own know-how, which may take a great deal of time and effort. Finally, while the discussion in this paper is focused on the compulsory licensing of trade secrets to facilitate access to COVID-19 vaccines, it may also be useful for any involuntary technology transfer of complex biological medicines, which are becoming prevalent in health care.

## 3. Vaccine technologies and their IP protection

### 3.1 What is a vaccine and how is it produced?

Vaccines are a critical tool for defeating the COVID-19 pandemic. They work by prompting the body’s immune system to recognize and beat the viruses and bacteria that attack it. Once vaccinated, the body is able to prevent illness by fighting off disease-causing organisms when exposed to them.^26^ Some vaccines, such as inactivated and protein-based vaccines, contain dead virus or tiny fragments of the disease-causing organism to trigger an immune response; others, such as adenovirus- and RNA-based vaccines, contain genetic material from the virus that triggers the production of virus proteins after injection and generates an immune response.^27^

There are currently several types of COVID-19 vaccines, including inactivated virus,^28^ live-attenuated vaccine,^29^ viral vector^30^ and the genetic approach (nucleic acid vaccine).^31^ The latter type of vaccine, messenger RNA (‘mRNA’), is currently the most challenging to produce because it is based on an entirely new technology^32^ for which there is a very limited manufacturing capacity and a shortage of expertise and essential components.^33^ As vaccines are complex biological products, their production involves a lengthy and complicated process of manufacture and control.^34^ While each vaccine has a unique manufacturing process, certain stages are common, including the propagation of active components, purification, formulation, fill and finish and sampling and testing.^35^ This production is challenging for various reasons and involves, among other things, complex processes, specialist knowledge and experience,^36^ as well as requiring appropriate manufacturing facilities.^37^ Moreover, while the manufacture of conventional drugs involves relatively simple chemical syntheses, biological products, such as vaccines, require highly specific standards and procedures for all steps of production.^38^

By way of illustration, the method required to make the mRNA vaccines currently supplied by Moderna and Pfizer-BioNTech is briefly outlined below.^39^ The process consists of six discrete steps as follows

*Step 1*: using an appropriate bacterial culture, produce the precise DNA sequence that needs to be transcribed into mRNA.

*Step 2*: in a bioreactor, using appropriate enzymes, produce the mRNA using the DNA from step 1.

*Step 3*: produce lipids with positively charged groups on them. Producing these at scale is a complex step.

*Step 4*: is the most complex step in the chain. It consists of combining the Step 2 mRNA and Step 3 lipids into lipid nanoparticles. This requires the production of a ‘…well-defined mix of solid nanoparticles with consistent mRNA encapsulation…’.^40^ This, in turn, requires a bespoke microfilter device that enables the manufacture of very precisely created nanoparticles. Such a device enables very precise mixing, flow rates, concentrations and temperature controls necessary to produce the end product.

*Steps 5 and 6*: consist in the fill and finish steps and distribution (in the case of the Pfizer-BioNTech vaccine, at very low temperatures) to the desired destinations.

While some of these steps and the constituents needed to make the vaccines are well known, the combination of steps and the technology (particularly at step 4) required to produce the end product are the result of extensive trial and error and are accompanied by extreme levels of testing at each stage to ensure consistency and purity of the product.

The combination of steps required, the method of production, the equipment (particularly at step 4) and the experience of the engineers controlling the process taken together constitute the kind of trade secret that, along with any patents protecting, say, the vaccine formula, creates all-round protection for the product and the process by which it is produced.

### 3.2 Vaccines and IP rights

Vaccines are protected by a range of IP rights.^41^ The most prominent IP right relevant for vaccines and vaccine-related technologies is patents. Patents allow pharmaceutical companies to control and protect the results of their research and development by bestowing exclusive rights upon their owners. The patent holder has the right to prevent others from using his or her invention and thus controls the manufacture, distribution and pricing of such products.^42^ In relation to vaccines, patents may protect, for example, their formulations, including the combination of medicinal components and devices for vaccine administration (eg, an injection delivery system or a capsule designed to release the product in a particular area of the human body).^43^

The other significant IP right relating to vaccines is trade secrets. Trade secrets broadly include all types of information that provide an economic or competitive advantage to their owner because the information is not generally known.^44^ These relate to their methods of manufacture (see, for instance, the example given above), although test data, specific (unpatented) medical formulae, cell lines, genomic information and other biological materials may also be protected as trade secrets. In addition, pharmaceutical companies consider results collected from clinical trials to be trade secrets.^45^ Such information is protected by data protection regulations^46^ and is based on the provisions of Article 39(3) TRIPS, which requires WTO members to protect test data submitted to regulatory authorities against unfair commercial use and disclosure.

## 4. Currently available mechanisms of compulsory licensing

In case of a refusal to voluntarily licence a vaccine technology, governments may need to rely on compulsory licensing. This section will explain the currently available mechanism of compulsory licensing of patents and why it is not sufficient for involuntary transfer of vaccine technologies.

### 4.1 Compulsory licensing of patents

To accelerate access to COVID-19 vaccines, one of the often-suggested mechanisms is compulsory licensing.^47^ As pharmaceutical companies are actively patenting the results of their research into COVID-19 vaccines,^48^ these exclusive rights to COVID-19 vaccines may, therefore, restrict or even block access to such a therapy. However, international laws contain specific mechanisms relating to compulsory licensing and government use for non-commercial purposes, which permit limiting the exercise of exclusive rights under the patent.

A compulsory licence is an authorization granted by a state authority that allows the person who receives it to use the invention without the agreement of the patent holder.^49^ This mechanism can be found in the TRIPS Agreement.^50^ Moreover, in 2001, the Doha Declaration on the TRIPS Agreement and Public Health^51^ confirmed that the granting of compulsory licences was one of the flexibilities under the TRIPS Agreement, which all WTO members have the right to use if necessary.^52^ This mechanism has been implemented in the majority of jurisdictions worldwide and may be relied upon to address public health needs.^53^ A specific type of compulsory licence is ‘government use’,^54^ under which the state authorises its *own use* of a patented product by granting authorization to a state agency or department or even to a private entity.^55^ This may be an effective tool as the government does not need to send a formal request to the patent holder and can act upon its own initiative to resolve public health matters.^56^ When relying on this mechanism, governments would not need to spend time on negotiating a licence, as required by TRIPS in relation to a normal compulsory licence, and can grant government use when necessary.^57^

Certain limitations apply to compulsory licensing. A compulsory licence can typically be granted in relation to existing patents.^58^ The mechanism cannot be applied to patent applications.^59^ As some of the COVID-19 technologies are new, patent applications are currently being filed and will be granted in the coming years.^60^ Until the time of the patent grant, this mechanism of compulsory licensing will not, therefore, be applicable. Thus, national IP laws may need to be amended to allow compulsory licensing of patent applications.

Moreover, while compulsory licensing of patents may be useful in improving access to certain medicines (eg, small-molecule medicines), this mechanism may not be effective in relation to biologics, such as vaccines, because their manufacturing technology may be protected by trade secrets. Unlike small-molecule drugs, which are easier for others to reverse engineer and reproduce without the need to know a specific manufacturing process, the knowledge on how to produce a complex biological therapy, such as a vaccine, may be critical.^61^ Some argue that in the area of vaccines ‘a manufacturing process is a product’.^62^ Therefore, without such knowledge, a compulsory licence of patents would be insufficient,^63^ and there is no obligation for patent owners to provide any additional information under a compulsory licence beyond what is included in a patent specification.^64^ Currently, however, there is no equivalent mechanism in IP laws for compulsory licensing of trade secrets similar to the compulsory licensing mechanism developed for patents.

Moreover, concerned about being subjected to compulsory licensing of patents, pharmaceutical companies may be inclined to rely even more on trade secrets.^65^ This has led some authors to argue that trade secrets can be considered ‘among the most powerful legal weapons against [the] public’.^66^ Currently, however, IP laws provide no mechanisms to force pharmaceutical companies to disclose their lifesaving COVID-19 vaccine technologies without their consent to voluntary sharing, (there are, however, some limited tools in other laws, as those, for instance, available under competition law discussed below).^67^ This results in a dependence of countries, both developed and developing, upon pharmaceutical companies and the inability of countries to protect public health promptly.^68^ This is even though research for most of the vaccines was heavily subsidized by public funding.^69^ Therefore, the development of a mechanism that would supplement compulsory licensing of patents and allow compulsory access to and transfer of trade secrets protecting COVID-19 vaccine technologies is urgently needed.

## 5. Is compulsory licensing of trade secrets consistent with international IP law?

### 5.1 Trade secret laws do not provide absolute protection

The extent to which trade secrets may be subject to enforced disclosure or legitimate use by third parties is regulated by national law. Taking EU countries as an example, the Trade Secrets Directive, which harmonizes national laws across the EU, does not provide protection against legitimate creation or discovery of trade secret information^70^ or the reverse engineering of a product in the public domain.^71^ The Directive also explicitly recognizes the

…application of [European] Union or national rules requiring trade secret holders to disclose, for reasons of public interest, information including trade secrets, to the public or to administrative or judicial authorities for the performance of their duties to those authorities.^72^

The ‘public interest’ is a flexible concept, but the EU Trade Secrets Directive does at least recognize that national governments may displace national trade secret laws where the public interest in, for example, the acquisition of life-saving technology takes precedence over the protection of a trade secret.

In the pharmaceutical field, a third party has the right to access certain information submitted as part of a marketing authorization dossier, including clinical trial data.^73^ For example, in the EU, the European Medicines Agency (‘EMA’) provides third parties with access to clinical trial data under Regulation 1049/2001/EC on access to documents and the EMA’s Policy 0070.^74^ These two policy instruments contain the right to access documents held by public authorities, including the EMA.^75^ Such access, however, is subject to exception in the event that the disclosure would undermine the commercial interests of a natural or legal person, including IP rights (the so-called commercially confidential information (‘CCI’)), unless there is an overriding public interest.^76^ In 2020, the Court of Justice of the European Union (‘CJEU’) issued several decisions in disputes, where originators sought to annul the EMA’s decisions to grant a third-party access to a document containing data submitted in the context of a marketing authorization application.^77^ The CJEU confirmed that there was no general presumption of confidentiality for clinical and toxicological study reports and upheld the General Court’s refusal to dismiss EMA’s decisions granting access.

However, there are no specific provisions in IP law either in the EU or the USA that allow compulsory access to trade secrets in order for them to be shared with competitors or the state.^78^ As a result, while a trade secret can be licensed voluntarily, a request for a licence could be denied.^79^ This already happened when several national manufacturers requested the licensing of the available vaccine technologies in a number of countries, which was denied by vaccine producers.^80^ For example, Biolyse, a Canadian manufacturer of cancer drugs, which is able to manufacture two million doses a month and thus has the potential to contribute to the global scale-up of vaccine manufacturing capacities, sought to manufacture and export the Johnson & Johnson (‘J&J’) adenovirus vaccine to developing countries. However, J&J refused to licence its technology to Biolyse.^81^

### 5.2 Compulsory licensing of trade secrets based on public interest

An important element of public interest is incorporated in trade secret laws. In the UK, for example, Lord Goff in the *Spycatcher* case^82^ said that there was a ‘public interest in the maintenance of confidence…[and]…the law will provide remedies for their protection’.^83^ A countervailing public interest defence against an action for breach of confidence, developed by the English courts, acknowledges that there are circumstances that may enable the recipient of confidential information to disclose that information to an appropriate person (a regulator) or, in some circumstances, to the media because the public interest recognizes the justification for doing so.^84^ That notion of public interest would not, however, stretch to providing justification for the enforced disclosure of trade secret technology by way of a compulsory licence.

The role of the public interest in trade secret law involves a balancing of the interests of trade secret holders against the public interest in disclosing trade secrets. While such a balancing exercise is typically undertaken when considering a defence against the unauthorised disclosure of trade secrets, there are isolated examples of the courts relying upon public interest to grant third parties access to trade secrets. In *Detroit Med. Ctr. v. GEAC Computer Sys., Inc*,^85^ for example, a case involving a proprietary computer software licensed to a hospital, the US court granted a preliminary injunction and forced the trade secret holder to provide confidential access to the hospital’s third-party service provider. When deciding whether to grant access to confidential information, the court undertook a balancing exercise and weighed, on the one hand, the public interest in protecting public health and, on the other hand, the interest in protecting confidential information. The court concluded that ‘the public’s interest in receiving adequate medical care outweighs its general interest in the performance of such [confidentiality] agreements’.^86^

It could be argued that in the case of compulsory licensing of trade secrets related to COVID-19 vaccines, there is an overarching public interest for disclosure of such trade secrets. Public interest disclosure would not presuppose public disclosure. On the contrary, it would require disclosure (or transfer) to another company, accompanied by a strict obligation of confidentiality. Therefore, such a mechanism is less damaging for the owner than the ‘public interest defence’ currently available in law. It is argued that the public interest argument in trade secret law should be extended from merely being relied upon as a defence against unauthorised disclosure to its use as a specific ground for granting a compulsory licence of trade secrets. This is especially important in a time of global pandemic, which serves as the perfect ground for invoking public interest.

### 5.3 Compulsory licensing of trade secrets and the TRIPS agreement

While Article 39 of the TRIPS Agreement may be considered to be the guiding world standard for the protection of trade secrets, it is useful to consider whether there are other elements of the TRIPS Agreement that may act to counterbalance the protection given to them.

Article 39 is drafted in terms of protection against unfair competition.^87^ It requires members to protect undisclosed information ‘[i]n the course of ensuring effective protection against unfair competition as provided in Article *10bis* of the Paris Convention (1967)’. The regime of unfair competition essentially protects against unfair commercial practices. The law of trade secrets, as set out in TRIPS, protects against *misappropriation* of trade secrets, which is actionable if the trade secrets were acquired *improperly* and are either used or disclosed or in violation of a duty to maintain confidentiality.^88^ Specifically, an acquisition of a trade secret by improper means occurs ‘if it was obtained through theft, bribery, misrepresentation, breach or inducement of a breach of a duty to maintain secrecy, or through espionage, including electronic espionage.’^89^

At the same time, the TRIPS Agreement and the Doha Declaration lay down important principles and objectives in relation to the protection of public health. In particular, Article 7 TRIPS provides that ‘[t]he protection and enforcement of intellectual property rights should contribute to the promotion of technological innovation and the *transfer and dissemination of technology*, … *in a manner conducive to social and economic welfare*, ….’ (emphasis added). In addition, Article 8 states that ‘Members may, in formulating or amending their laws and regulations, *adopt measures necessary to protect public health’* and that ‘[a]ppropriate measures may be needed to prevent the abuse of intellectual property rights by right holders or the resort to practices which …. *adversely affect the international transfer of technology*’ (emphasis added). Finally, paragraph 4 of the Doha Declaration states that ‘the TRIPS Agreement does not and should not prevent members from taking measures to protect public health’ and that it ‘can and should be interpreted and implemented in a manner supportive of WTO members “right to protect public health and, in particular, to promote access to medicines for all”’.^90^ Therefore, the interpretation of these provisions may lay down the grounds for a compulsory licensing of trade secrets under TRIPS to ensure the protection of public health, especially during the COVID-19 pandemic, by facilitating the production of vaccines through an international technology transfer.^91^

Moreover, the TRIPS Agreement contains no specific exclusions that would prevent compulsory licensing of trade secrets. In particular, while TRIPS has a provision on compulsory licensing of patents, it expressly prohibits compulsory licensing of trade marks. Article 21 states that it is ‘understood that the compulsory licensing of trade marks shall not be permitted’. It could be argued that had the drafters intended to exclude this mechanism from being applied to trade secrets, they would have explicitly stated so. Instead, the TRIPS Agreement remains silent on this issue, thus, arguably, leaving this matter for national legislation. Therefore, this could be construed as allowing governments to issue compulsory licensing of trade secrets when required, including for the protection of public health.

## 6. Potential grounds for granting a compulsory licence of trade secrets protecting COVID-19 vaccines

As can be seen from the above discussion, compulsory licensing of trade secrets is arguably in line with the TRIPS Agreement. While there are currently no specific provisions in the law regarding compulsory licensing of trade secrets, the fundamental basis for their granting may stem from the states’ obligations under national and international laws to protect public health.^92^ During the pandemic, this could be granted under laws that mandate the protection of citizens against the pandemic. A number of countries have recently enacted emergency laws for this reason.

For example, France has implemented emergency law n° 2020–290 of 23 March 2020 to deal with the COVID-19 epidemic that introduced a new article L.3131–15 into the Public Health Code. This article allows the Prime Minister, when a state of health emergency is declared and for the sole purpose of guaranteeing public health: (i) to order the requisition of *all goods* and services necessary to fight the health disaster and of any person necessary for the operation of these services or the use of these goods and (ii) to take all ‘*measures to make available to patients appropriate medicines for the eradication of the health disaster*’ (emphasis added).^93^ This provision provides broad powers to the Prime Minister enabling him/her to issue a compulsory licence/government use of any patent or patent application,^94^ as well as trade secrets that relate to a vaccine. This is because this provision generally refers to ‘goods’ and ‘all measures’ and, therefore, goes beyond protection by patents.^95^

Moreover, the US Defense Production Act (‘DPA’)^96^ allows the President to require businesses to prioritize contracts that promote the national defence.^97^ It has been argued that, based on the DPA, the President has the power to require pharmaceutical companies that have developed and are producing COVID-19 vaccines to share information and data needed to facilitate increased production.^98^

Based on these various legal provisions, as well as the public interest considerations mentioned above, compulsory licensing of trade secrets may be possible by governmental orders (similar to ‘government use’ of patents), which would oblige vaccine producers to disclose and provide access to all the information, including trade secrets, required to manufacture a vaccine. However, while some emergency laws discussed above may provide a basis for granting a compulsory licence of trade secrets, it is advisable to implement this mechanism more specifically in the IP law, thus supplementing compulsory licensing of patents with a similar mechanism for trade secrets. This will ensure that governments can facilitate access to medicines effectively by granting compulsory licensing of patents and trade secrets. This is important because more and more medicines, including vaccines, are characterized as complex biologics, protected not only by patents but also by a significant number of trade secrets. Moreover, without such an additional mechanism, compulsory licensing of patents may become a ‘shallow’ and ineffective tool and, hence, the flexibilities envisaged in the TRIPS Agreement that were implemented to balance strong proprietary patent rights would have no effect. Therefore, since TRIPS is silent about compulsory licensing of trade secrets, countries are free to implement such provisions in their national laws. It would also be desirable to harmonize this instrument at an international level by including relevant provisions in the TRIPS Agreement.

## 7. Trade secrets and licensing

This section will explain in more detail what trade secrets are and the particularities of trade secrets licensing compared to other IP rights, which will lay down the grounds for the discussion of compulsory licensing of trade secrets in the next section.

### 7.1 What are the characteristics of trade secrets that potentially make them so valuable in the field of life sciences?

A trade secret represents the foundation of every IP right, or, as the European Commission described it in a report analysing the field of trade secrets, ‘every intellectual property right starts life as a trade secret’.^99^ An as-yet unpatented invention, the idea in a designer’s head for a new innovative product or the plot for a new film or novel all have value based on their confidential status. Once the invention takes the form of a patent application or the idea becomes recorded, the status of the right changes but until the application is published or the idea becomes available, their value rests on confidentiality.

Trade secrets, like other IP rights, are national rights, protected under the laws of the country in which the owner (or controller—see below) is based or of the country where an infringement action is pursued.^100^ Some countries do so by unfair competition laws; others by a combination of tort, contract and employment statutes or codes. US trade secret laws are based on State law overlaid by a degree of uniformity in the Uniform Trade Secrets Act^101^ and Federal laws in the shape of the Economic Espionage Act 1996 and the Defend Trade Secrets Act 2016. The UK and other countries that follow the English common law base their protection predominantly on case law and the development of the breach of confidence action.^102^

Signatory countries to the TRIPS Agreement are obliged to provide protection to trade secrets as part of their national laws. Article 39 of the TRIPS Agreement is as follows

1. In the course of ensuring effective protection against unfair competition as provided in Article 10bis of the Paris Convention (1967), Members shall protect undisclosed information in accordance with paragraph 2 and data submitted to governments or governmental agencies in accordance with paragraph 3.
2. Natural and legal persons shall have the possibility of preventing information lawfully within their control from being disclosed to, acquired by, or used by others without their consent in a manner contrary to honest commercial practices so long as such information

- is secret in the sense that it is not, as a body or in the precise configuration and assembly of its components, generally known among or readily accessible to persons within the circles that normally deal with the kind of information in question;
- has commercial value because it is secret; and
- has been subject to reasonable steps under the circumstances, by the person lawfully in control of the information, to keep it secret.

The reference to a ‘manner contrary to honest commercial practices’ in paragraph 2 means

…at least practices such as breach of contract, breach of confidence and inducement to breach, and includes the acquisition of undisclosed information by third parties who knew, or were grossly negligent in failing to know, that such practices were involved in the acquisition.

Although drafted in terms of protection against unfair competition, Article 39 makes it clear that a ‘manner contrary to honest commercial practices’ will include actions such as breach of contract (for example, breach of a standard non-disclosure agreement) or breach of confidence, the latter defining the legal action by which trade secrets are enforced in the UK and other common law countries.

The TRIPS definition borrows its ‘reasonable steps’ language from the US Uniform Trade Secrets Act 1985. The definition has also been used in its entirety in the EU Trade Secrets Directive,^103^ introduced in 2016 to harmonize trade secrets laws in EU countries.

That the EU chose to harmonize national laws in this way reflects another feature of trade secrets: they are becoming more, rather than less, important. In a survey carried out by an international law firm in 2017, CEOs in companies across a range of business sectors said that they considered their trade secrets to be more important than their patents, copyrights or trade marks.^104^ This is in line with the detailed analysis carried out by the European Commission before the EU Trade Secrets Directive was introduced and is consistent with a number of other studies carried out in recent years.^105^

### 7.2 Trade secret licensing

To answer the question: how compulsory licensing of trade secrets might be achieved as a matter of law and of practice, it is necessary to consider what a trade secret licence is and how it, in its various guises, operates.

A licence conveys no proprietary interest in the underlying IP. It simply authorises the licensee to do something by contract, which, in the absence of the licence, would be an infringement of the licensor’s rights. There is relatively little material about trade secret licensing.^106^ A licence of a trade secret has some clear similarities with other IP licences. But it also has some fundamental differences. If a licensor licenses its patent to a licensee, which later commits a breach of the licence, entitling the licensor to terminate the licence, the licensor still has its patent and can find an alternative licensee. By contrast, if a trade secret licensor licenses its rights to a licensee, which breaches the confidentiality provisions of the licence by deliberately or inadvertently disclosing the secret to the public domain, the licensor has, in effect, nothing left to commercialize. Since the commercial value of the trade secret is its secrecy, and that secrecy disappears, so too does the substance of the licensable right. The licensor may have a significant claim for damages against the guilty licensee, but that may hardly compensate it for the loss of its licensing business.

This, in turn, reflects a significant difference between trade secrets and other IP rights. Trade secrets rely for their value on being kept secret. Once secrecy disappears, so too does the value of the right. That explains why, in many countries, trade secret protection is not a function of property rights but of the relationship of confidence between the ‘owner’ and the recipient of the information.^107^

A trade secret licence will tend to be one of four types of licence

- *A hybrid patent—trade secret licence*. This is usually called a patent and know-how licence and recognizes the fact that much patented technology is more usefully applied if you know how to put the technology into practical effect.^108^ So, the patent element might protect the key technology, but the trade secret (or know-how) element represents the most effective way of implementing it, by, for example, operating a machine or mixing components in a particular way.
  There are two key aspects to a hybrid licence that are worth mentioning, which are also features of the other licences discussed below. The first is the ability to define what the trade secret consists of. The ability to define the secret is one of the most difficult but important aspects of trade secret law. It is important for licensor and licensee alike. A licensee is entitled to know what it is entitled to use (and what it is paying for) and, hence, what it must be careful to protect by keeping it secret. Definition is also important to distinguish any improved method devised by the licensee and to which it may lay claim and, perhaps, licence back to the licensor. The second is a comprehensive confidentiality provision designed to impose strict obligations on the licensee to protect the secrecy and value of the information. Confidentiality terms will range from maintaining password-protected access to documents to disclosure on a need-to-know basis to manufacturing process operatives. Without these, there would be no effective way for the licensor to control or police how its information was being used.^109^
- *A ‘pure’ trade secret licence* in which the technology is the trade secret information. In practice, these are relatively rarely encountered. Their effectiveness depends to a great extent on the kind of detailed confidentiality provisions outlined above, designed to give the licensor maximum control over how the licensee uses and protects the information in question.
- *A Technical Assistance Agreement*. This is a combination of a licence and training by the licensor of the licensee’s operatives on how to use the technology concerned. In such a licence, in addition to defined technology and detailed confidentiality terms, there will be usually quite extensive provisions on how and where instruction and training of the licensee’s operatives will take place, in what language and for how long. Training will almost always be at the licensee’s expense. A technology assistance agreement recognizes that it can take a great deal of time to understand, operate and maintain highly complex processes (such as vaccine technology). In many cases, it will simply be insufficient to hand over some instruction manuals and hope that the licensee’s operatives are able to get the process to work. They need to be shown how the technology works in practice, hence the fact that agreements of this sort are also often called ‘show-how’ licences. This essential attribute of technical assistance is recognized in the wider context of technology transfer from developed to less developed economies
  Transfer of know how is largely a question of training and teaching sometimes accomplished through formal educational programmes and international exchanges, but usually through informal learning and on-the-job training.^110^
- *A ‘turn-key’ agreement.* This is an altogether more complex arrangement, consisting of a combination of licence, training and constructing the plant or process line that encompasses the technology concerned. The additional element—actually constructing the physical plant or production line, gives the licensor some additional degree of protection, particularly if certain aspects of the plant or line are ‘black box’ components that conceal the secret technology and to which the licensee’s operative has no access.

There is one common feature to these licensing transactions, which is not so common in other IP licensing arrangements, and that is how and why the licensor chooses its licensee. The relationship between licensor and licensee is assumed to be a purely commercial one, in which the licensor’s interest is in earning an income from allowing the licensee to use the technology under licence. The same is true of other IP transactions. But the fact that the IP concerned—trade secrets—is different from other IP rights because of its unique fragility to being disclosed makes the relationship between licensor and licensee a different one. As previously explained, a licensor entitled to terminate a licence with a licensee still has its IP and can find another licensee. But a licensor of trade secrets, which have been, deliberately or inadvertently, disclosed to the world at large, has nothing left to licence.

This, in turn, explains why a trade secret licensor must make a different assessment of its licensee: is the licensee likely to be a trustworthy business into whose hands the licensor is prepared to put its secret information? Due diligence on the licensee thus assumes a higher level of significance than in other cases. The licensor will ask itself a number of questions about the licensee

- What is its status—a limited liability company, a partnership, a limited liability partnership and, in any of these, how long has it been established?
- What do its published or disclosed accounts say about its financial strength?
- What experience does its management have in operating a complex technology-based business?
- What controls and processes does it have in place to maintain security of the information against internal and external threat?
- In particular, can the licensee create a ‘culture of confidentiality’ in the workplace to enhance the protection given to the licensor’s trade secrets?^111^
- What is its track record of compliance with licences-in?
- In which country is it based and what legal mechanisms (particularly early-stage injunctions and the like) are available to enable the licensor, should the need arise, to enforce its contractual terms against the licensee.

In short, can the licensee be trusted with the licensor’s trade secret information?

## 8. The practicalities and difficulties associated with compulsory licensing of trade secrets related to COVID-19 vaccines

The manufacture of a complex vaccine is unlike the manufacture of a small-molecule drug where the beneficiary of a compulsory licence does not need access to details of the manufacturing process in order to produce an identical product.^112^ A licence of the patent would be sufficient to do this.^113^ It is also not necessary to duplicate a potentially patent-protected manufacturing process to ensure an identical product, and a third-party licensee may use an alternative method of manufacture to produce the desired end product. By contrast, vaccines are complex biologics and their production requires a set of specific knowledge and expertise, including knowledge about the manufacturing process extending to the use of specific items of equipment unique to the process (possibly designed by the vaccine manufacturer itself).

Any government intent on putting a compulsory licence into the hands of a licensee must, as a first step, identify a potentially suitable licensee. That licensee must at least have a plant, equipment and some degree of expertise in this kind of manufacturing. The licensee would need to ‘set up, calibrate and test equipment, and train scientists and engineers to run it’.^114^ The licence must identify the scope of technology transfer, including the scope of information necessary for production and, as the section above on trade secret licensing made clear, much would depend on the need for technical assistance or ‘show how’ to enable the licensee to make effective use of the technology. As a leading article in The Times newspaper put it: ‘publishing blueprints to allcomers would be like handing out a vastly complex recipe without the skills or access to ingredients required to execute it’.^115^

### 8.1 Elements of a compulsory licence of trade secrets

There are a number of elements that a compulsory licence of trade secrets must contain, which are akin to those that would typically be included in a voluntary licensing agreement. In general, a voluntary IP licence includes, among other things, identification of the licensor and licensee, specification of the rights licensed, type of licence (exclusive, sole or non-exclusive), restrictions imposed on the licensee and remuneration, usually based on royalty payments. In addition, there are measures to ensure confidentiality and probably warranties from the licensor as to entitlement to grant the licence and from the licensee on the quality of products manufactured, sometimes backed by an indemnity designed to protect the licensor from product liability claims. The licence will include termination provisions, the usual range of ‘boilerplate’ provisions dealing with issues such as force majeure and, crucially, a dispute resolution mechanism and governing law clause.^116^ A trade secret licence normally contains additional ‘provisions that define the area of technology with precision, establish a confidential legal relationship between the parties, furnish proprietary information for a specific purpose only, oblige the recipient to hold information in confidence, and spell out exceptions to secrecy obligations’.^117^

Where then would a compulsory trade secret licence differ from a voluntary licence agreement? The following are likely to be the key areas of difference

#### 8.1.1 Parties to the compulsory licence

Given the element of compulsion, the licence is likely to be structured as a three-party licence, i.e. compulsory licensor, compulsory licensee and the government imposing the order to grant the licence. The government as a third party would also be necessary to act as guarantor of the licensee’s obligations to maintain the confidentiality of the technology.

#### 8.1.2 Type of licence

It would be a non-exclusive, non-sublicensable and non-assignable licence so that the licensor could use and licence the technology to other licensees and the government concerned could compulsorily licence it to other companies to accelerate vaccine production.

#### 8.1.3 What is being licensed?

A compulsory licence must specify precisely what is being licensed, ie, ‘what specific technology and proprietary information attaches to the transfer’.^118^ This is one of the key provisions and must be clearly defined in order to ensure full access to everything the licensee needs to successfully manufacture the vaccine in question. If the process is adequately described and defined in documentary form—formulae, production methods, standard operating procedures, plant design blueprints and so on—the licence will set out the array of documents needed to equip the licensee with what it needs to undertake manufacturing.

The real difficulty emerges when access to documents is simply not enough to enable the licensee to manufacture the vaccine and what is needed is the technical assistance (‘show how’) of the licensor. Usually, this will take the form of the physical presence of the licensor’s scientific or technical staff to supervise or assist in the setting up of the plant, its operation and the training of its staff. How would the licence document prescribe this often vital aspect of a trade secret licence? How would the licence impose an obligation on the licensor to detach key members of its staff and send them, possibly to a foreign country and possibly for months on end to undertake this work? Is this akin to the granting of a mandatory injunction to the licensor compelling it to make its staff, possibly against their will, attend the licensee’s premises?

More to the point, how could this be enforced? Assume a compulsory licence imposed by a government in country A against a licensor-technology owner in country B, which requires an element of ‘show how’ training (which is currently the case for most developing countries). If the putative licensor refused to comply with country A’s order, some form of reciprocal enforcement of a foreign government or foreign court order would be necessary in country B to oblige the licensor to comply. The reciprocal recognition and enforcement of foreign judgments depends on whether countries, sometimes as a block such as EU countries, or on an individual basis, have concluded treaties to recognize and enforce another country’s court orders. For many countries no such reciprocal system exists.

And even then, the imposition of a requirement to force employees from the employer’s workforce to attend a process plant in country A to provide training and supervision of operatives there is difficult to envisage. Of course, one outcome of the COVID-19 pandemic has been an explosion of remote working, so the assistance of Teams or Zoom or similar technology could, at least in part, provide a solution to the problem. Nonetheless, if the ‘show how’ element of the licence required the physical presence of the licensor’s personnel, then the enforcement, on a cross-border basis, of that obligation poses real problems to which there is no immediately obvious solution.

Other aspects of the enforcement of a compulsory licence also need to be considered. What, for example, would be the position if the compulsory licensee claimed that the supervision or training provided by the licensor were not of a sufficiently high standard to equip its technicians with the skills to operate the process? What would be the licensee’s remedy? Not to terminate the licence or even claim damages for breach because that would not solve the alleged problem. Instead, it would demand specific performance of the licence, enforced, presumably, by a local court or by the government party obliging the licensor’s technical staff to do a better job. Again, this assumes ease of enforcement by way of reciprocal enforcement of a judgment or government order, which, as pointed out above, in many judicial systems will simply not be available.

#### 8.1.4 The grant and prohibitions

The grant clause lies at the heart of any licence since it defines the scope of what the licensee can do with the licensor’s rights. Use within the scope of the grant is non-infringing use. Use outside is both infringing and in breach of contract. The grant clause in a vaccine production and supply licence would therefore be to manufacture, store, sell and distribute the vaccine.

#### 8.1.5 Improvements

During the use of the licensed trade secrets, the licensee might make certain improvements to the technology, eg, to the manufacturing process. The licence would need to deal with the ownership of the licensee’s improvements. Given that the licence would be a compulsory one, it would seem reasonable to assign any improvement back to the licensor, but local competition law might prohibit that.

#### 8.1.6 Duration of the licence

The length of the licence should cover the period of the pandemic and potentially could be extended as long as the government considered it necessary to utilize this technology for the protection of public health, for example, for repeat or supplementary vaccinations to maintain protection.

#### 8.1.7 Obligations and standards of confidentiality

It is axiomatic that confidentiality and its preservation is at the heart of any trade secret licence, which is why, in normal circumstances, a licensor would need to be assured by its due diligence that the licensee was a fit and proper person to act as a licensee and custodian of the licensor’s trade secret material. A compulsory licensee would be chosen by the government concerned and hence any element of choice by the licensor would be displaced.

A compulsory licence could impose an express obligation on the government to exercise its best efforts to select a suitable licensee and to indemnify the licensor against any breach of the licensee’s confidentiality obligations. The interpretation of ‘best efforts’ will differ from one legal system to another. Governments in some countries may not even have the luxury of an array of suitably equipped or qualified licensees to choose from. And what would the government’s position be if the chosen licensee simply flouted the terms of the licence by disclosing the technology, thereby destroying its secrecy and value? As guarantor of the licensee’s obligations, the government’s position to protect the interests of the licensor seems clear. On the other hand, there may be little a licensor could do to prevent a government from evading its obligations by reference to some form of sovereign immunity.

The licence would need to impose strict obligations to introduce and observe security for the information. These provisions would include, for example, secure IT and document access systems, concealed production areas, the use of confidentiality provisions in operatives’ employment contracts and so on.

How the licensee would enforce confidentiality provisions against employees is, of course, a major issue in any such licence. It is well recognized that employees are the main source of trade secret misappropriation. This is not the place for a discussion of what employees can and cannot take when they leave an employer’s employment.^119^ That will always be a matter for local law. But if the licensee’s employees are the main threat to the licensor’s trade secrets, the licensor is at the mercy of two things. The first is what local law says (if it says anything at all) on the freedom of an employee to change jobs and to use skill and experience acquired in previous employment. There is no bright line (at least in, eg, English law) between an employer’s (or in this case, a licensor’s) trade secrets and an employee’s skill and experience. Local law might well struggle with a case, particularly one where the only effective remedy would be an early-stage injunction, to restrain an employee moving from one job to another for an increased salary or a higher-level position. The second issue, related to the first, is whether it would be possible to oblige the licensee to take action against a former employee threatening to use or disclose the licensor’s trade secrets. The licensee might have perfectly good reasons not to do so, not least if local legal advice was that any such case would fail. Even if the compulsory licensor had taken the precaution of having a direct contractual relationship with process operatives (for example, by way of a non-disclosure agreement), the same question of local law enforcement would be likely to arise.

#### 8.1.8 Royalties

Since this is a compulsory licence, the royalties paid to the licensor should be set by the government at a level that, on the one hand, would not impede the rationale of such a compulsory licence and, on the other hand, would adequately compensate the owner for the use of its technology. The royalties could be calculated following the approaches taken to calculate royalties with respect to compulsory licensing of patents. This is typically a rate of 2–4 per cent based on generic product price.^120^

#### 8.1.9 Termination

An important provision in the compulsory licence would be the effective date of termination of such a licence. The licence should be terminated when the circumstances due to which it was granted cease to exist, ie, upon eradication of the pandemic. In addition, because trade secrets must be protected and kept confidential at all times, the obligation of confidentiality must continue after the agreement is terminated and, therefore, the licensee may be required to protect trade secrets from disclosure for an additional period of time.^121^ The reservations expressed above about the possible effectiveness of confidentiality obligations during the licence term apply with added emphasis here. Additionally, the rights and obligations of the parties upon termination or expiration of the licence may include the obligation of the licensee to return, destroy and cease to use the proprietary information and all the related documents.^122^ It is questionable how effective this would be if the licence had enabled the licensee to construct an entire plant or production line and whether the obligation would mean, in effect, the destruction of something that had allowed local expertise and employment to be developed.

#### 8.1.10 Warranties and penalties

Given that the licence would be compulsory, it would seem unreasonable for the government or licensee to demand any warranties from the licensor on the effectiveness of the technology or its end result—the vaccine produced. By contrast, it would seem reasonable for the licensor to be protected by a warranty from the licensee that it would hold the licensor harmless from any product liability claim arising from the licensee’s use of the technology to manufacture and use of the vaccine concerned.

#### 8.1.11 Governing law and dispute resolution

It is foreseeable that a government-imposed licence would involve the imposition of a local law and forum provision. This is unlikely to be of much comfort to a compulsory licensor, particularly if local law and courts were unfamiliar with complex technology-based licences or lacked the essential means to enforce, as discussed above, say, the confidentiality provisions that are at the heart of the licence.

This brief summary provides some examples of how difficult the implementation and enforcement of a compulsory trade secrets licence might be for licensor and licensee alike. But it is the licensor’s position that is the most vulnerable. There is no doubt that the licence could include elaborate provisions to protect the licensor’s rights. That is not the point. The real issue lies in cross-border enforcement of confidentiality provisions under local law, which may have little or no experience of trade secrets or effective remedies to prevent their misuse or disclosure. The fact remains that once the information leaks to a competitor or to the public, there is no simple way it can be recaptured. Therefore, to make compulsory licensing of trade secrets work effectively, some jurisdictions, along with implementing this mechanism, may also need to reform their enforcement regime to address the challenges discussed above.

## 9. Suggested wording of a compulsory vaccine technology transfer

Given the reservations expressed in the previous section, the question remains: would it be feasible to introduce such a compulsory licence? The answer is a qualified yes, and a template for such a licence exists. The US Federal Trade Commission (‘FTC’) recently imposed a compulsory licence on a pharmaceutical company in the *Mallinckrodt Ard Inc. (Questcor Pharmaceuticals)* case,^123^ according to which the company had to share its technology related to a biological drug, adrenocorticotropic hormone, including patents and trade secrets, with a designated third-party licensee.

The licence imposed on the defendant technology owner was in the form of a perpetual, irrevocable, fully paid up, sublicensable, assignable, exclusive licence to commercialize the licensor’s pharmaceutical product in a defined field and territory. The licence extended to a licence (on the same terms) to use the licensor’s trade marks and its medical and regulatory information. The licence dealt with the problem of putting the licensor’s trade secrets into the licensee’s hands in the following way (in the following extract, the licensee is identified as the ‘Sublicensee’)

C. … Defendants shall provide the… Sublicensee with a full and complete copy of all tangible documentation and records embodying the Licensed IP and Manufacturing Technology^124^ in the Defendants’ possession or control, which if in electronic form shall be readily useable with off-the-shelf commercially available software and equipment. Defendants shall deliver all such documentation and records to the… Sublicensee in good faith, in a timely manner (*i.e.* as soon as practicable, avoiding any delays in transmission of the respective documents and records), and in an organized and comprehensive manner that ensures completeness and accuracy and that fully preserves the usefulness of such documents and records. Pending complete delivery of all such documents and records to the…. Sublicensee, Defendants shall provide the…. Sublicensee and the Monitor (if any has been appointed) with access to all such documents and records and employees of the Defendants who possess or are able to locate such information for the purposes of identifying the books, records, and files directly related to the Licensed IP and Manufacturing Technology and facilitating the delivery in a manner consistent with this Order.

G. Upon the…. Sublicensee’s request, Defendants shall provide access to the … Sublicensee to any manufacturing site (whether or not owned or controlled by the Defendants; *provided, however* that, for any manufacturing site not owned or controlled by Defendants, Defendants are only required to provide access to the extent that Defendants have access and as permitted under any agreement(s) with the manufacturing site) that Defendants use to manufacture [the product] and make such arrangements with any Third Party necessary to permit that access for the purposes of evaluating and learning the manufacturing process…and discussing the process with Persons involved in the manufacturing process (including, without limitation, use of equipment and components, manufacturing steps, time constraints for completion of steps, methods to ensure batch consistency), pharmaceutical development, and validation of the manufacturing of [the product] at that facility.

H. … Defendants shall provide reasonable access to Defendants’ personnel to provide instructions and answer questions regarding the application of the Licensed IP and Manufacturing Technology (to the extent known by Defendants).

The reference to the ‘Monitor’ is to an independent third party appointed by the FTC to oversee the performance by the licensor of its obligations under the licence, including the provision of documents and granting of access to personnel to provide manufacturing technology information.

This compulsory licence was granted in very different circumstances dealing with an anti-trust violation. The granting of a compulsory licence to make COVID 19 vaccine technology available has the public interest at its core. But the public interest must also have regard to the interests of the licensor technology owner, the latter having potentially spent a great deal of time and money on creating commercial value in its confidential technology. How is the licence meant to protect the licensor’s rights to its technology against misappropriation by an employee of the licensee or against unauthorised access caused by poor security management by the licensee?

The role of the monitor, introduced in the *Mallinckrodt* case, shows that an independent third party might play a significant role in overseeing access to and protection of the licensor’s technology, in essence to oversee fair play in what would be an enforced contractual relationship far removed from the normal commercial technology licensing arrangement between commercial parties. In particular, some of the problem issues relating to the enforcement of cross-border obligations considered in Section 8 above might be dealt with by the intervention and supervision of a trusted third-party monitor.

## 10. Further issues with compulsory licensing of trade secrets

When granting a compulsory licence of a vaccine, some further challenges may arise. One of the barriers that also needs to be overcome when issuing a compulsory licence on a medicine or vaccine relates to data and marketing exclusivity that protects clinical test data submitted by the originator to the relevant regulator. Such exclusivity aims to prevent other pharmaceutical companies from relying on such data during the term of protection to obtain a marketing authorization for their generic or biosimilar version of the originator’s medicine. For example, the EU pharmaceutical regulation provides for 8 years of data exclusivity, plus 2 years of market exclusivity during which generic companies can apply for their marketing authorization (but they cannot market their generics during this 2-year period).^125^ This EU exclusivity regime covers both small molecules and biological products.^126^ After the expiration of such exclusivity, generic companies can rely on the originator’s data submitted to the regulator, thus avoiding the need to duplicate extensive clinical trials to prove that their generic version of a brand-name drug is safe and effective. Generic companies only need to show that their generic version is bioequivalent to an originator’s already approved product. Also, applicants for biosimilar medicines (generic biological medicines) can refer to data submitted by the originator.^127^ They are required to ‘demonstrate through comprehensive comparability studies with the “reference” biological medicine that: (a) their biological medicine is highly similar to the reference medicine, notwithstanding natural variability inherent to all biological medicines; and (b) there are no clinically meaningful differences between the biosimilar and the reference medicine in terms of safety, quality and efficacy’.^128^ In the context of compulsory licensing, this means that such exclusivity would prevent a compulsory licensee from obtaining a marketing authorization for its vaccine. Several authors have suggested that such exclusivity should be waived to allow the licensees under compulsory licences to obtain their marketing authorizations before it expires.^129^ Moreover, a biosimilar manufacturer is typically required to conduct more testing and submit more data to demonstrate the similarity of its product to the approved original biological medicine than a generic manufacturer of a traditional generic medicine. This may present an additional barrier to increasing vaccine manufacture and supply. Therefore, while Article 39 TRIPS requires providing regulatory data exclusivity, the regime must be reconsidered by removing such a barrier when there is a need to protect public health as mandated by the key principles in the TRIPS Agreement discussed above.

## Conclusions

The COVID-19 pandemic has brought significant challenges to the world community and revealed the inability of the current system of access to medicines to effectively address the devastating effect of this pandemic at the global level. To defeat this pandemic, an accelerated production of COVID-19 vaccines and their equitable distribution worldwide are urgently needed. This is not an easy task, as there is currently not enough manufacturing capacity to produce the billions of doses that are needed to swiftly inoculate the entire world population. In addition, there is another, perhaps even more serious, hurdle—to accelerate vaccine production, access to vaccine technologies is required. However, such technologies are protected by an array of IP rights that are owned by pharmaceutical companies. To remove this IP barrier, various proposals have been put forward, including voluntary technology pools (C-TAP and other initiatives), compulsory licensing and the TRIPS IP waiver. However, each of these solutions has a major drawback: the process of vaccine manufacture is protected by trade secrets and there exists no mechanism to oblige pharmaceutical companies to share them.

This paper suggests that to make compulsory licensing of patents or the IP waiver work, an additional mechanism of compulsory licensing of trade secrets is required. It is argued that there is an overarching public interest for the disclosure of trade secrets related to COVID-19 vaccines. This mechanism will also be in line with the TRIPS Agreement, which, on the one hand, does not explicitly prohibit compulsory licensing of trade secrets and, on the other hand, mandates that its principles should be construed in a manner supportive of WTO members’ rights to protect public health. Without such an additional mechanism, the flexibilities envisaged in the TRIPS Agreement in the form of compulsory licensing of patents, which were implemented to balance strong proprietary patent rights, will have no effect.

It is believed that the proposal for a new mechanism of compulsory licensing of trade secrets coupled with a discussion on the content of such licences, challenges that would need to be addressed and the potential wording of such a licence would provide useful guidance to governments on how to make their compulsory technology transfer mechanisms more effective. Importantly, this mechanism would be relevant both in case of the adoption of the TRIPS IP waiver, as well as if such a mechanism were not agreed upon and thus WTO members needed to rely on the existing compulsory licensing mechanism related to patents. More fundamentally, as more and more drugs on the market are complex biologics and are thus protected by trade secrets, the suggested mechanism would remain relevant after the pandemic.

## Footnotes

1 Global leaders unite in urgent call for international pandemic treaty (*WHO press release*, 30 March 2021). Available at https://www.who.int/news/item/30-03-2021-global-leaders-unite-in-urgent-call-for-international-pandemic-treaty (accessed 21 May 2021).

2 ‘COVID-19 vaccine tracker’ Available at https://covid19.trackvaccines.org/vaccines/ (accessed 21 May 2021) (‘As of 15 May 2021, 15 different vaccines approved worldwide’).

3 ‘Covid-19 Treatment and Vaccine Tracker’, available at https://milken-institute-covid-19-tracker.webflow.io/#vaccines_intro; ‘Draft landscape and tracker of COVID-19 candidate vaccines’, available at https://www.who.int/publications/m/item/draft-landscape-of-covid-19-candidate-vaccines (accessed 21 May 2021).

4 Aisling Irwin, ‘What it Will Take to Vaccinate the World Against COVID-19. A Special Report Outlines the Challenges - From Unleashing the Power of mRNA Vaccines, to the Battle for Temporary Intellectual Property Relief’ (*Nature* 25 March 2021). Available at https://www.nature.com/articles/d41586-021-00727-3 (accessed 21 May 2021).

5 ‘Director-General’s Opening Remarks at One Shared World Event’ (*WHO*, 5 May 2021), available at https://www.who.int/director-general/speeches/detail/director-general-s-opening-remarks-atone-shared-world-event (accessed 21 May 2021); Mary B Marcus, ‘Ensuring Everyone in the World Gets a COVID Vaccine’ (*Duke Global Institute* 20 January 2021), available at https://globalhealth.duke.edu/news/ensuring-everyone-world-gets-covid-vaccine (accessed 21 May 2021). (‘As of mid-January, more than 7 billion vaccine doses had been purchased globally and the lion’s share—4.2 billion doses—have gone to high-income countries. While high-income countries represent only 16% of the world’s population, they currently hold 60% of the vaccines for COVID-19 that have been purchased so far. Canada tops the list having purchased enough vaccine to cover more than five times their population.’).

6 Irwin (n 4); see also ‘Companies warn that 9 out of 10 people in poor countries are set to miss out on COVID-19 vaccine next year’ (*Oxfam Press Release*, 9 December 2020), available at https://www.oxfam.org/en/press-releases/campaigners-warn-9-out-10-people-poor-countries-are-set-miss-out-covid-19-vaccine (accessed 21 May 2021); Owen Dyer, ‘Covid-19: Many Poor Countries Will See Almost No Vaccine Next Year, Aid Groups Warn’ (2020) 371 *BMJ* m4809, available at https://www.bmj.com/content/371/bmj.m4809 (accessed 21 May 2021).

7 WTO, WIPO, WHO, ‘Promoting Access to Medical Technologies and Innovation Intersections between Public Health, Intellectual Property and Trade’ (2012), available at https://www.wipo.int/edocs/pubdocs/en/global_challenges/628/wipo_pub_628.pdf (accessed 21 May 2021); Report of the United Nations Secretary-General’s High-Level Panel on Access to Medicine, ‘Promoting Innovation and Access to Health Technologies’ (2016) 22, available at http://www.unsgaccessmeds.org/final-report (accessed 21 May 2021).

8 Sara Eve Crager, ‘Improving Global Access to New Vaccines: Intellectual Property, Technology Transfer and Regulatory Pathways’ (2014) 104 *American Journal of Public Health* e85 (suggesting the creation of an ‘Intellectual Property, Technology and Know-How’ (‘IPTK’) Bank which would merge process information and regulatory information on a single platform. The IPTK Bank would dispense non-exclusive licences similar to the Medicines Patent Pool).

9 Alexis K Juergens and Leslie P Francis, ‘Protecting Essential Information About Genetic Variants and Trade Secrets: A Problem for Public Policy’ (2004) 5 *Journal of Law and the Biosciences* 682–705.

10 Tara Nealey, Ronald M Daignault, and Yu Cai, ‘Trade Secrets in Life Science and Pharmaceutical Companies’ (2015) 5 *Cold Spring Harbor Perspectives in Medicine* a020982.

11 WHO ‘COVID-19 Technology Access Pool’. Available at https://www.who.int/initiatives/covid-19-technology-access-pool (accessed 21 May 2021).

12 ibid; Viviana Muñoz Tellez, ‘The COVID-19 Pandemic: R&D and Intellectual Property Management for Access to Diagnostics, Medicines and Vaccines’ (2020) the South Centre Policy, Brief No 73.

13 Michael Safi, ‘WHO platform for pharmaceutical firms unused since pandemic began’ (*The Guardian* 22 January 2021). Available at https://www.theguardian.com/world/2021/jan/22/who-platform-for-pharmaceutical-firms-unused-since-pandemic-began (accessed 21 May 2021).

14 Muhammad Zaheer Abbas, ‘Treatment of the Novel COVID-19: Why Costa Rica’s Proposal for the Creation of a Global Pooling Mechanism Deserves Serious Consideration?’ (2020) 1 *Journal of Law and the Biosciences* 1.

15 Communication from India and South Africa, ‘Waiver from Certain Provisions of the Trips Agreement for the Prevention, Containment and Treatment of Covid-19’ (2 October 2020) IP/C/W/669. Available at https://docs.wto.org/dol2fe/Pages/SS/directdoc.aspx?filename=q:/IP/C/W669.pdf&Open=True (accessed 21 May 2021).

16 WTO Agreement on Trade-Related Intellectual Property Rights, Marrakesh Agreement Establishing the World Trade organisation, Annex 1C, 15 April 1994, in World Trade organisation, The Legal Texts: The Results of the Uruguay Round of Multilateral Trade Negotiations 321 (1999), as amended on 23 January 2017 (hereinafter ‘TRIPS Agreement’).

17 Communication from the African Group, The Plurinational State of Bolivia, Egypt, Eswatini, Fiji, India, Indonesia, Kenya, The LDC Group, Maldives, Mozambique, Mongolia, Namibia, Pakistan, South Africa, Vanuatu, the Bolivarian Republic of Venezuela and Zimbabwe, ‘Waiver From Certain Provisions Of The TRIPS Agreement For The Prevention, Containment And Treatment Of COVID-19. Revised Decision Text’ (21 May 2021) IP/C/W/669/Rev.1. Available at https://docs.wto.org/dol2fe/Pages/SS/directdoc.aspx?filename=q:/IP/C/W669R1.pdf&Open=True? (accessed 23 June 2021).

18 ‘Statement from Ambassador Katherine Tai on the Covid-19 Trips Waiver’ (*United States Trade Representative Press Release* 5 May 2021). Available at https://ustr.gov/node/10649 (accessed 21 May 2021).

19 Communication From the European Union to the Council for TRIPS, ‘Urgent Trade Policy Responses to the Covid-19 Crisis: Intellectual Property’ (Brussels 4 June 2021), available at https://trade.ec.europa.eu/doclib/docs/2021/june/tradoc_159606.pdf (accessed 23 June 2021); Julian Borger and Patrick Wintour, ‘US-Germany rift as Berlin opposes plan to ditch Covid vaccine patents’ (*The Guardian*, 6 May 2021), available at https://www.theguardian.com/world/2021/may/06/us-germany-rift-covid-vaccine-patent-waivers (accessed 21 May 2021); ‘A patent waiver on COVID vaccines is right and fair’ (*Nature*, 23 May 2021), available at https://www.nature.com/articles/d41586-021-01242-1 (accessed 23 June 2021).

20 John Smeaton and Lydia Harriss, ‘Manufacturing COVID-19 vaccines’ (*UK Parliament* 14 January 2021). Available at https://post.parliament.uk/manufacturing-covid-19-vaccines/ (accessed 21 May 2021); Derek Lowe, ‘COVID_19: Myths of Vaccine Manufacturing’ (*Science Translational Medicine Blog*, 2 February 2021). Available at https://blogs.sciencemag.org/pipeline/archives/2021/02/02/myths-of-vaccine-manufacturing (accessed 21 May 2021).

21 Salla Sariola, ‘Intellectual Property Rights need to be subverted to ensure global vaccine access’ (2021) 6 *BMJ Global Health* e005656; see also Lawrence O Gostin, Safura Abdool Karim, and Benjamin Mason Meier, ‘Facilitating Access to a COVID-19 Vaccine through Global Health Law’ (2020) 48 *The Journal of Law, Medicine and Ethics* 622.

22 David S Levine, ‘Covid-19 Trade Secrets and Information Access: An Overview’ (*InfoJustice* 2020), available at http://infojustice.org/archives/42493 (accessed 21 May 2021); David S Levine, ‘Trade Secrets and the battle against Covid’ (2020) 15 *JIPLP* 849 (where it is argued that ‘…voluntary trade secret information sharing and/or compulsory trade secret licensing could be extended to any number of other areas where trade secrecy has been a barrier to more rapid information sharing and innovation, from climate change to energy production, to the next pandemic.’).

23 ibid; Karen Walsh et al., ‘Intellectual Property Rights and Access in Crisis’ (2021). Available at https://link.springer.com/article/10.1007/s40319-021-01041-1 (accessed 21 May 2021) (‘If a [patent] licence is granted, be it voluntary or compulsory…there are remaining non-IPR access barriers relating to technology transfer, data and market exclusivity, and know how, which are necessary to make the invention work. It is important to note that trade secrets may also be operating in the background.’); similarly, see Aisling McMahon, ‘Patients, access to health and COVID 19 – the role of compulsory and government-sue licensing in Ireland’ (2020) 7 *NI Legal Quarterly* 331, 338 (‘In the vaccine context…having a compulsory licence over the patent will not necessarily *on its own* enable a third party to produce a similar version…This information may not be disclosed by the patent holder thereby requiring the third party to develop this knowledge…and this may be difficult and/or take considerable time…such issues merely support the argument that, once a compulsory licence is issued on a patented invention, patent holders should also disclose related information around the working of that invention, such as know- how and trade secret information.’).

24 See, eg, McMahon (n 23); Olga Gurgula and Wen Hwa Lee, ‘COVID-19, IP and Access: Will the Current System of Medical Innovation and Access to Medicines Meet Global Expectations?’ (2021) forthcoming in the *Journal of Generic Medicines*, available at https://ssrn.com/abstract=3771935 (accessed 21 May 2021); Kayvan Bozorgmehr et al., ‘Free Licensing of Vaccines to End the COVID-19 Crisis’ (2021) 397 *The Lancet* 1261, 1261; Katrina Perehudoff, Ellen ‘t Hoen and Pascale Boulet, ‘Overriding Drug and Medical Technology Patents for Pandemic Recovery: A Legitimate Move for High-Income Countries, too’ (2021) 6 *BMJ Global Health* e005518. doi:10.1136/.

25 See references in (n 22 and 23); Suma Athreye, ‘Vaccine Platforms and Limited Global Production Capacity: What is to be Done?’ (IPKat, 2021). Available at https://ipkitten.blogspot.com/2021/05/vaccine-platforms-and-limited-global.html (accessed 21 May 2021).

26 WHO ‘How do Vaccines Work?’ (8 December 2020). Available at https://www.who.int/news-room/feature-stories/detail/how-do-vaccines-work (accessed 21 May 2021).

27 Smeaton and Harriss (n 20).

28 WHO ‘The different types of COVID-19 vaccines’ (12 January 2021) (‘The first way to make a vaccine is to take the disease-carrying virus or bacterium, or one very similar to it, and inactivate or kill it using chemicals, heat or radiation.’). Available at https://www.who.int/news-room/feature-stories/detail/the-race-for-a-covid-19-vaccine-explained (accessed 21 May 2021).

29 ibid (‘A live-attenuated vaccine uses a living but weakened version of the virus or one that’s very similar’).

30 Robert Weber, ‘Explaining Johnson & Johnson’s, AstraZeneca’s new COVID-19 Vaccines’ (*The Ohio State University Wexner Medical Center* 2 March 2021). Available at https://wexnermedical.osu.edu/blog/explaining-johnson-johnson-astrazeneca-vaccines (accessed 21 May 2021) (‘Viral vector vaccines use a modified, harmless version of a different virus as a vector, or carrier, to deliver immunity instructions to cells in the body. The body then follows those instructions to build an immune response to the intended virus … The virus vector being used in the Johnson & Johnson and AstraZeneca vaccines is an adenovirus, a common type of virus that typically causes mild cold symptoms when it infects someone’).

31 WHO ‘The Different Types of COVID-19 Vaccines’ (12 January 2021) (n 28) (‘… a nucleic acid vaccine just uses a section of genetic material that provides the instructions for specific proteins, not the whole microbe. DNA and RNA are the instructions our cells use to make proteins. In our cells, DNA is first turned into messenger RNA, which is then used as the blueprint to make specific proteins. A nucleic acid vaccine delivers a specific set of instructions to our cells, either as DNA or mRNA, for them to make the specific protein that we want our immune system to recognize and respond to. The nucleic acid approach is a new way of developing vaccines. Before the COVID-19 pandemic, none had yet been through the full approvals process for use in humans…’).

32 Anthony Komaroff, ‘Why are mRNA Vaccines so Exciting?’ (*Harvard Health Blog*, 18 December 2020). Available at https://www.health.harvard.edu/blog/why-are-mrna-vaccines-so-exciting-2020121021599 (accessed 21 May 2021).

33 Irwin (n 4); see, however, Priti Patnaik, ‘Views From a Vaccine Manufacturer: Q&A - Abdul Muktadir, Incepta Pharmaceuticals; Pandemic Treaty Action’ (30 March 2021). Available at https://genevahealthfiles.substack.com/p/views-from-a-vaccine-manufacturer (accessed 21 May 2021).

34 Vaccines Europe, ‘How are Vaccines Produced?’. Available at https://www.vaccineseurope.eu/about-vaccines/how-are-vaccines-produced (accessed 21 May 2021).

35 Smeaton and Harriss (n 20).

36 ibid.

37 Hilde Stevens et al., ‘Vaccines: Accelerating Innovation and Access. Global Challenges Report’ (*WIPO*, 2017) 14. Available at https://www.wipo.int/publications/en/details.jsp?id=4224 (accessed 21 May 2021).

38 ibid (‘Vaccines, especially more sophisticated products, are subject to relatively lengthy and costly quality control requirements. … Controls must exist at every stage of the manufacturing process to ensure the safety and quality of the completed batch. They include tests for physicochemical properties, such as pH and osmolality, component identity and stability analyses for antigens, excipients and adjuvants, microbiological testing for sterility, concentration, and potency testing and animal-based testing for toxicity. The tests imposed on vaccine manufacturers may vary according to the national legislation and requirements on the part of national regulatory agencies’ (internal citations deleted)).

39 This explanation is largely taken from Lowe (n 20); see also the explanation given at Elizabeth Weise and Karen Weintraub, ‘A COVID-19 Vaccine Life Cycle: From DNA to Doses’ (*USA Today*, 2021). Available at https://eu.usatoday.com/in-depth/news/health/2021/02/07/how-covid-vaccine-made-step-step-journey-pfizer-dose/4371693001/ (accessed 21 May 2021).

40 Lowe (n 20).

41 Stevens et al. (n 37) 19 (‘Various IP rights are relevant for vaccines and vaccine-related technologies, including patents, trademarks, copyrights, and trade secrets.’).

42 Carlos M Correa, ‘Guide for the Granting of Compulsory Licenses and Government Use of Pharmaceutical Patents’ (2020) 107 *The South Centre Research Paper* 13; McMahon (n 23) 322.

43 Stevens et al. (n 37) 19.

44 For a detailed discussion on the main characteristics of trade secrets, see Section 7.

45 Aaron S Kesselheim and Michelle M Mello, ‘Confidentiality Laws and Secrecy in Medical Research: Improving Public Access To Data On Drug Safety’ (2020) 26 *Health*  *Affairs* 483–91. Available at https://www.healthaffairs.org/doi/abs/10.1377/hlthaff.26.2.483?journalCode=hlthaff (accessed 21 May 2021).

46 Stevens et al. (n 37) 20 (‘In most sectors, companies can protect commercially sensitive data through trade secrecy laws, but the requirement for vaccine manufacturers to disclose data to regulators puts them at a competitive disadvantage. Clinical test data protection thus ensures that competitors cannot gain regulatory approval and enter the market via reliance on an innovator’s test data before the innovator has had an opportunity to recoup the costs of compiling it.’); Kristina Lybecker, ‘When Patents aren’t Enough: Why Biologics Necessitate Data Exclusivity Protection’ (2014) 40 *William Mitchell Law Review* 1427–42.

47 For an extensive literature review related to compulsory licensing, see Danielle Navarro and Marcela Vieira, ‘Research Synthesis: Compulsory Licensing’ (2021). Graduate Institute Geneva. Available at https://www.knowledgeportalia.org/compulsory-licensing (accessed 21 May 2021).

48 Carlos M Correa, ‘Expanding the Production of COVID-19 Vaccines to Reach Developing Countries’ (2021) *The*  *South Centre Policy Brief* 91, available at https://www.southcentre.int/wp-content/uploads/2021/04/PB-92.pdf (accessed 21 May 2021); Communication from South Africa, ‘Examples of IP Issues and Barriers in Covid-19 Pandemic’ (*WTO*, 23 November 2020), IP/C/W/670, available at https://docs.wto.org/dol2fe/Pages/SS/directdoc.aspx?filename=q:/IP/C/W670.pdf&Open=True (accessed 21 May 2021); Cynthia Koons, ‘The Vaccine Scramble Is Also a Scramble for Patents’ (*Bloomberg*, 12 August 2020), available at https://www.bloomberg.com/features/2020-covid-vaccine-patent-price/; Achal Prabhala and Ellen ‘t Hoen, ‘We’ll find a treatment for coronavirus—but drug companies will decide who gets it’ (*The Guardian*, 15 April 2020) (‘…there is every indication that treatments for coronavirus may soon emerge, the mere fact of their existence is no guarantee that people will be able to access them. In fact, Covid-19 is more likely to end in the same way that every pandemic ends: treatments and vaccines will be buried in a thicket of patents – and pharmaceutical companies will ultimately make the decisions about who lives and who dies’…‘Remdesivir, a medicine developed for Ebola by the biotechnology company Gilead, has major patents across the world that last until 2038’) all accessed 21 May 2021.

49 Medicines Law & Policy: Research and resources on intellectual property and health. Available at https://medicineslawandpolicy.org/tools/ (accessed 21 May 2021).

50 Article 31 of the TRIPS Agreement.

51 Declaration on the TRIPS Agreement and Public Health (14 November 2001), Doc.WT/MIN(O1)/DEC/2 (20 November 2001) (hereinafter ‘the Doha Declaration’).

52 ibid, Sub-paragraph 5 (b) ‘Accordingly and in the light of paragraph 4 above, while maintaining our commitments in the TRIPS Agreement, we recognize that these flexibilities include: … b. Each Member has the right to grant compulsory licenses and the freedom to determine the grounds upon which such licenses are granted.’

53 Correa (n 42) 13.

54 See, eg, Section 55 of the Patents Act 1977 (UK law contains such a concept as ‘crown use’). See also ‘Germany, UK, USA: Are Patent Exceptions the Cure To COVID-19?’ (*JDSUPRA*, 15 April 2020), available at https://www.jdsupra.com/legalnews/germany-uk-usa-are-patent-exceptions-35625/ (accessed 21 May 2021); François Pochart et al., ‘Compulsory licenses granted by public authorities: an application in the Covid-19 crisis in France?’ (*Kluwer Patent Blog*, 23 April 2020), available at http://patentblog.kluweriplaw.com/2020/04/23/compulsory-licenses-granted-by-public-authorities-an-application-in-the-covid-19-crisis-in-france-part-1/ (accessed 21 May 2021).

55 Medicines Law & Policy: Research and resources on intellectual property and health. Available at https://medicineslawandpolicy.org/tools/ (accessed 21 May 2021).

56 Correa (n 42) 13.

57 ibid; Article 31 TRIPS ‘Other Use Without Authorization of the Right Holder’ (‘Where the law of a Member allows for other use of the subject matter of a patent without the authorization of the right holder, including use by the government or third parties authorised by the government, the following provisions shall be respected: (b) such use may only be permitted if, prior to such use, the proposed user has made efforts to obtain authorization from the right holder on reasonable commercial terms and conditions and that such efforts have not been successful within a reasonable period of time. This requirement may be waived by a Member in the case of a national emergency or other circumstances of extreme urgency or in cases of public non-commercial use’). See also Gurgula and Lee (n 24).

58 Pochart et al. (n 54).

59 ibid; Correa (n 48).

60 Pochart et al. (n 54).

61 McMahon (n 23) 338; Crager (n 8) e87 (arguing that for a successful vaccine access strategy ‘[a]ccess to manufacturing process information protected by trade-secret law, as well as access to technology and know-how held by the innovator company, will likely be necessary’).

62 For a discussion see Nicola Searle, ‘The process may (or may not) be the product: trade secrets and COVID research’ (*The*  *IPKat*, 3 August 2020). Available at https://ipkitten.blogspot.com/2020/08/the-process-may-or-may-not-be-product.htm (accessed 21 May 2021).

63 McMahon (n 23) 338; Crager (n 8) e87 (arguing that ‘although patent protection remains the major barrier to the production of affordable small-molecule generics, access to trade-secret - protected information and know-how present major additional obstacles to generic production of vaccines’).

64 Stevens et al. (n 37) 22 (arguing that due to this ‘compulsory licenses may not be as cost-effective as voluntary methods of medical technologies procurement, particularly when licenses are issued for local production in low-income countries. In fact, manufacturers are likely to be disincentivized from sharing such information under these circumstances.’).

65 Sebastian Alvarado, ‘Chile: Trade Secrets as a Response to Compulsory Licensing’ (*Mondaq*, 23 July 2020). Available at https://www.mondaq.com/trade-secrets/968600/trade-secrets-as-a-response-to-compulsory-licensing (accessed 21 May 2021).

66 Levine, ‘Covid-19 Trade Secrets and Information Access: an Overview’ (n 22).

67 ibid (‘Government regulators can also run into challenges getting access to trade secrets, especially absent clear statutory mandates for access.’).

68 Safi (n 13) (explaining that the WHO COVID-19 Technology Access Pool (C-TAP) has attracted zero contributions since it was established in May 2020); Grace Ren, ‘Progress on COVID-19 Technology Pool Inches Along as Sister Initiative to Pool Vaccine Procurement Accelerates’ (*Health Policy Watch*, 25 September 2020). Available at https://healthpolicy-watch.news/progress-on-covid-19-technology-pool-inches-along-as-sister-initiative-to-pool-vaccine-procurement-accelerates/ (accessed 21 May 2021) (‘unlike the COVAX Facility, which has received broad industry support, the COVID-19 Technology Access Pool has been dismissed by the pharmaceutical industry, which holds much of the rights to the technology, data, and research that the Pool would aim to more freely distribute’).

69 Bozorgmehr et al. (n 24) (‘These pharmaceutical companies have benefited greatly from huge sums of public funding for research and development and advance purchase commitments, amounting to between US$2.2 billion and $4.1 billion (by 1 February 2021) from Germany, the UK, and North America combined’).

70 Article 3 (1) (a) of the Directive (EU) 2016/943 of the European Parliament and of the Council of 8 June 2016 on the protection of undisclosed know-how and business information (trade secrets) against their unlawful acquisition, use and disclosure (hereinafter ‘the EU Trade Secrets Directive’).

71 ibid at Art 3 (1) (b).

72 ibid at Art 2(b).

73 GIPC, ‘Clinical Data and Disclosure Policies: The European Union, Member States, and International Best Practices’ (2015). Available at https://www.theglobalipcenter.com/clinical-data-and-disclosure-policies-the-european-union-member-states-and-international-best-practices/ (accessed 21 May 2021).

74 EMA, ‘European Medicines Agency policy on Publication of Clinical data for Medicinal Products for Human use’ (*POLICY/0070*, 21 March 2019). Available at https://www.ema.europa.eu/en/documents/other/european-medicines-agency-policy-publication-clinical-data-medicinal-products-human-use_en.pdf (accessed 21 May 2021).

75 Daria Kim, ‘Transparency Policies of the European Medicines Agency: Has the Paradigm Shifted?’ (2017) 25 *Medical Law Review* 456.

76 Article 4 of Regulation No 1049/2001.

77 C-175/18 P *PTC Therapeutics International Ltd v EMA* [2020] ECLI:EU:C:2020:23; C-178/18 P *MSD Animal Health Innovation and Intervet International v EMA Agency* [2020] ECLI:EU:C:2020:24; Case C-576/19 P *Intercept Pharma and Intercept Pharmaceuticals v EMA* [2020] ECLI:EU:C:2020:873.

78 Levine, ‘Covid-19 Trade Secrets and Information Access: an Overview’ (n 22).

79 ibid.

80 Ashleigh Furlong, ‘Big Vaccine Makers Reject Offers to Help Produce More Jabs’ (*POLITICO*, 14 May 2021). Available at https://www.politico.eu/article/vaccine-producers-reject-offers-to-make-more-jabs/ (accessed 21 May 2021) (it was reported that ‘Biolyse in Canada, Incepta in Bangladesh, Teva in Israel and Bavarian Nordic in Denmark have all asked to assist in the manufacture of vaccines. As yet, none has a deal’); McMahon (n 23) 322 (‘Whilst many patent-holders have shown willingness to offer favourable licensing terms for COVID-19 health-related technologies, there is no legal requirement for rights-holders to do so, and others in future may not.’).

81
*See* Arianna Schouten, ‘Canada Based Biolyse Pharma Seeks to Manufacture COVID-19 Vaccines for Low Income Countries, May Test Canada’s Compulsory Licensing for Export Law’ (*KEI*, 12 March 2021). Available at https://www.keionline.org/35587 (accessed 21 May 2021).

82
*Attorney-General v Guardian Newspapers Ltd* (No 2) [1990] 1 AC 109.

83 ibid [281].

84 Starting life as the ‘iniquity defence’ in *Gartside v Outram* (1856) L.J. Ch 113, it has developed (not without its critics) into a more expansive defence based on public interest grounds. See *Initial Services v Putterill* [1967] 3 All ER 145 and *Lion Laboratories v Evans* [1984] 2 All ER 417.

85
*Detroit Medical Center v. GEAC COMPUTER SYSTEMS*, 103 F. Supp. 2d 1019 (E.D. Mich. 2000).

86 ibid [1024].

87 International Chamber of Commerce, ‘Protecting Trade Secrets - Recent EU and US Reforms’ 2019, 8, available at https://iccwbo.org/content/uploads/sites/3/2019/04/final-icc-report-protecting-trade-secrets.pdf (accessed 21 May 2021); Tanya Aplin, ‘Right to Property and Trade Secrets’ in Christopher Geiger (ed), *Research Handbook on Human Rights and Intellectual Property* (Edward Elgar 2015) 422.

88 Karl F Jorda, ‘Trade Secrets and Trade-Secret Licensing’ in Anantole Krattiger et al. (ed) *Intellectual Property Management in Health and Agricultural Innovation: A Handbook of Best Practices* (Concept Foundation 2007) 1047.

89 ibid.

90 Levine, ‘Covid-19 Trade Secrets and Information Access: An Overview’ (n 14).

91 Kung-Chung Liu, ‘The Need and Justification for a General Competition-Oriented Compulsory Licensing Regime’ (2012) 43 *IIC**—**International Review of Intellectual Property and Competition Law* 683; H. Xue, ‘Enforcement for Development: Why not an Agenda for the Developing World?’ in X Li and C Correa (eds) *Intellectual Property Enforcement—International Perspectives* (Edward Elgar 2009) 148.

92 The right to health is a fundamental human right. It was first recognized in the 1946 WHO constitution, which stated that ‘the enjoyment of the highest attainable standard of health is one of the fundamental rights of every human being without distinction of race, religion, political belief, economic or social condition’. This right was also included in the 1948 Universal Declaration of Human Rights and in the 1966 International Covenant on Economic, Social and Cultural Rights. It was also recognized or referred to in other international human rights treaties. See WHO, ‘The right to health. Factsheet N 31’ (2008). Available at https://www.who.int/gender-equity-rights/knowledge/right-to-health-factsheet/en/ (accessed 21 May 2021) (‘The right to health is relevant to all States: every State has ratified at least one international human rights treaty recognizing the right to health. Moreover, States have committed themselves to protecting this right through international declarations, domestic legislation and policies, and at international conferences.’).

93 Pochart et al. (n 54).

94 Bird & Bird, ‘Q&A on the use of patented products and processes without authorisation of patent holder National compulsory licence laws’ (2020). Available at https://www.twobirds.com/∼/media/pdfs/in-focus/coronavirus/lsh-tracker/bird—bird-compulsory-licensing-all.pdf?la=en&hash=BE1D4031E433E11C25CF4C82AC4E8CA30C83A691 (accessed 21 May 2021).

95 ibid.

96 The Defense Production Act of 1950.

97 Public Citizen, ‘Report: How Biden Can Shore Up Global, U.S. Production of COVID-19 Vaccines’ (8 December 2020). Available at https://www.citizen.org/news/report-how-biden-can-shore-up-global-u-s-production-of-covid-19-vaccines/ (accessed 21 May 2021).

98 Elizabeth Warren ‘Sen. Warren and Rep. Porter Urge Full Use of Defense Production Act to Bolster the Nation’s Vaccine Supply as COVID-19 Deaths Hit Daily Records’ (15 January 2021). Available at https://www.warren.senate.gov/oversight/letters/sen-warren-and-rep-porter-urge-full-use-of-defense-production-act-to-bolster-the-nations-vaccine-supply-as-covid-19-deaths-hit-daily-records (accessed 21 May 2021).

99 European Commission, ‘Final Study on Trade Secrets and Confidential Business Information in the Internal Market’ (2013). Available at https://file:///C:/Users/olgag/Downloads/130711_final-study_en.pdf (accessed 21 May 2021).

100 Trade secret laws differ from country to country and no attempt is made here to provide detailed source material. See Trevor Cook, *Trade Secret Protection: A Global Guide* (Globe Law and Business 2016); also ‘Trade Secrets’ (WIPO). Available at https://www.wipo.int/tradesecrets/en/ (accessed 21 May 2021). A good perspective on the area is provided in SK Sandeen and EA Rowe, *Trade Secrets and Undisclosed Information* (Edward Elgar 2014).

101 Uniform Trade Secrets Act 1979 (amended in 1985).

102
*Coco v A.N.Clark (Engineering) Ltd* [1969] RPC 41. Generally: Tanya Aplin et al., *Gurry on Breach of Confidence* (2nd edn OUP 2012). The UK has implemented the EU Trade Secrets Directive—see the Trade Secrets (Enforcement etc) Regulations SI 2018/597.

103 Article (n 70).

104 Baker and Mckenzie, ‘The Board Ultimatum: Protect and Preserve’ (2017). Available at https://www.bakermckenzie.com/-/media/files/insight/publications/2017/trade-secrets (accessed 21 May 2021).

105 Andrew Schwartz, ‘The Corporate Preference for Trade Secrets’ (2013) 74 *Ohio State Law Journal* 623; Katherine Linton, ‘The Importance of Trade Secrets’ (2016) *Journal of International Commerce and Economics* 1.

106 Jorda (n 88); Dennis Unkovic, *The Trade Secrets Handbook* (Prentice Hall 1985); John Hull, ‘Trade Secret Licensing: the Art of the Possible’ (2009) 4 *JIPLP* 203; John Hull, ‘The Licensing of Trade Secrets and Know How’ in Jacques de Werra (ed) *Research Handbook on Intellectual Property Licensing* (Edward Elgar 2013); Maxim Tsotsorin, ‘Practical Considerations in Trade Secret Licensing’ (2012), available at https://papers.ssrn.com/sol3/papers.cfm?abstract_id=2334060 (accessed 21 May 2021).

107 The EU Trade Secrets Directive (n 70) specifically avoided designating trade secrets as property rights, which is why the directive avoids using the term ‘owner’ and instead defines the party entitled to enforce a trade secret as the ‘holder’, meaning one who lawfully controls the trade secret—Art 2 (2).

Noel Byrne and Amanda McBratney, *Licensing Technology: Negotiating and Drafting Technology Transfer Agreements* (3rd edn Jordans 2005) 6.6.

In a recent English Court of Appeal judgment, Arnold LJ makes the point that ‘…the doctrine of misuse of confidential information…is all about the control of information’. *Shenzhen Senior Technology Material v Celgard* [2020] EWCA Civ 1293 at [23].

Srijit Mukherjee and Sudipta Bhattacharjee, ‘Technology Transfer and the Intellectual Property Issues Emerging from it – an Analysis from a Developing Country’ (2004) 9 *Journal of Intellectual Property Law* 260, 261.

R Mark Halligan and Richard F Weyand, *Trade Secret Asset Management: An Executive’s Guide to Information Asset Management, Including Sarbanes-Oxley Accounting Requirements for Trade Secrets* (Aspatore 2006) Chapter 10.

112 Crager (n 8) e86 (‘Biologic drugs, a category that includes vaccines, are generally produced by living cells and are significantly larger and structurally more complex than small-molecule drugs’).

113 McMahon (n 23) 338.

114 Liz Szabo et al., ‘Why Even Presidential Pressure Might Not Get More Vaccine to Market Faster’ (*KHN*, 26 January 2021). Available at https://khn.org/news/article/ramping-up-covid-vaccine-production-could-take-months-even-with-bidens-best-tool-to-pressure-companies/ (accessed 21 May 2021).

115
*The Times* 7 May 2021.

116 Mark Anderson and Victor Warner, *Technology Transfer* (4th edn Bloomsbury Professional 2020) Chapter 2.

117 Jorda (n 88) 1055.

118 Tsotsorin (n 106) 5.

119 Magdalena Kolasa, *Trade Secrets and Employee Mobility—In Search of Equilibrium* (CUP 2018).

120 James Love, ‘Remuneration Guidelines for Non-Voluntary Use of a Patent on Medical Technologies’ (2005) World Health organisation and United Nations Development Programme. Available at https://apps.who.int/iris/bitstream/handle/10665/69199/WHO_TCM_2005.1_eng.pdf?sequence=1&isAllowed=y (accessed 21 May 2021).

121 Tsotsorin (n 106) 10.

122 ibid.

123
*FTC v Mallinckrodt Ard Inc*, ‘Stipulated Order for Permanent Injunction and Equitable Monetary Relief’, Case Number: 1:17-Cv-120 EGS (20 January 2017, US District Court for the District of Columbia). Available at https://www.ftc.gov/system/files/documents/cases/stipulated_order_for_permanent_injunction_mallinckrodt.pdf (accessed 21 May 2021).

124 ibid (‘According to the decision the “Manufacturing Technology” means all technology, trade secrets, know-how and proprietary information in each case to the extent necessary for the manufacture, validation, packaging, release testing, stability and/or shelf life of [the product] and/or the Drug Substance, including the …drug product formulations and/or other records.’).

125 Directive 2004/27/EC on the Community code relating to medicinal products for human use [2004] OJ L136/34. In the USA, the Biosimilar Price Competition and Innovation Act (BPCIA), enacted as part of the Patient Protection and Affordable Care Act (Public Law 111–148) on 23 March 2010, has created an abbreviated licensure pathway for biological products shown to be biosimilar to or interchangeable with an FDA-licensed biological reference product. The term of exclusivity for biologics in the USA is 12 years in total (including 4 years before an application for a biosimilar may be submitted to the FDA and 8 additional years before an application may be approved).

126 See Medicines Law & Policy, ‘Data Exclusivity in the European Union: Briefing Document’ (2019). Available at https://medicineslawandpolicy.org/wp-content/uploads/2019/06/European-Union-Review-of-Pharma-Incentives-Data-Exclusivity.pdf (accessed 29 July 2021).

127 ibid.

128 EMA, ‘Biosimilar medicines: marketing authorisation’. Available at https://www.ema.europa.eu/en/human-regulatory/marketing-authorisation/biosimilar-medicines-marketing-authorisation (accessed 29 July 2021).

129 Ellen Hoen et al., ‘Data Exclusivity Exceptions and Compulsory Licensing to Promote Generic Medicines in the European Union: A Proposal for Greater Coherence in European Pharmaceutical Legislation’ (2017) 10 *Journal of Pharmaceutical Policy and Practice* 19; Correa (n 42).

